# Potential of fungi isolated from the dumping sites mangrove rhizosphere soil to degrade polythene

**DOI:** 10.1038/s41598-019-41448-y

**Published:** 2019-03-29

**Authors:** Manisha K. Sangale, Mohd. Shahnawaz, Avinash B. Ade

**Affiliations:** 1 Department of Botany, Savitribai Phule Pune University, Pune, 411007 Maharashtra India; 2 Present Address: Department of Botany, S. M. Joshi College Hadapsar, Malwadi, Hadapsar, Pune, Maharashtra 411028 India; 3 Present Address: Plant Biotechnology Division, CSIR-Indian Institute of Integrative Medicine, Canal Road Jammu, Jammu, 180001 Jammu and Kashmir India

## Abstract

Polythene is the most widely used plastic around the globe. Among the total plastic waste generated, polythene contributes the maximum share (64%). Various strategies/methods are being utilized to deal with the increasing rate of plastic waste, but among all the methods, bioremediation is regarded as the ecofriendly and widely accepted method. In the current investigation, we have attempted to discover the elite polythene deteriorating fungi (isolated from the rhizosphere soil of *Avicennia marina*). From 12 different eco-geographical locations along the West Coast of India, total 109 fungal isolates were recorded. The polythene deteriorating fungi were screened at varied pH (3.5, 7 and 9.5) based on changes in weight and tensile strength of the treated polythene at ambient temperature with continuous shaking for 60 days. BAYF5 isolate (pH 7) results in maximum reduction in weight (58.51 ± 8.14) whereas PNPF15 (pH 3.5) recorded highest reduction in tensile strength (94.44 ± 2.40). Surprisingly, we have also reported weight gain, with highest percent weight gain (28.41 ± 6.99) with MANGF13 at pH 9.5. To test the reproducibility of the results, the elite polythene degrading fungal isolates based on weight loss and reduction in tensile strength were only used for repetition experiment and the results based on the reduction in tensile strength were found only reproducible. Polythene biodegradation was further confirmed using Scanning Electron Microscopy (SEM) and Fourier Transform Infrared Spectroscopy (FTIR) analysis. The most efficient polythene deteriorating fungal isolates were identified as *Aspergillus terreus* strain MANGF1/WL and *Aspergillus sydowii* strain PNPF15/TS using both morphological keys and molecular tools.

## Introduction

The word plastic is originated from the root word ‘plastikos’ (‘grow’ or ‘form’: able to be molded into different shapes) of the Greek language^1^. It is a polymer, made up of high molecular weight (petrochemicals), long chain of hydrocarbons^2^. Plastic in various forms tender services in our day-to-day life from our kitchen to industry level^3–5^ and thus increase its demand around the globe. The production of plastic is doubled annually, and was estimated as 250 million tons in 2008^5^. As per the report, the highest plastic consumer in the world is Asia (35%) followed by North America (26%), Western Europe (23%), Japan (6%) and India (5%)^5^. Due to the various beneficial properties of the plastic viz. stability, durability (mechanical and thermal property), the utilization of the plastic is at its peak and its demand is continuously increasing^6–8^. The synthetic plastic is non-biodegradable^9^ and/or having very slow or least rate of degradation, e.g. polythene needs about 1000 years to degrade under natural environment^5,10^. Mueller^11^ reveled that micro-organisms are unable to degrade plastic due to their short term presence in the environment and therefore during evolution microorganisms failed to design elite enzymes to degrade plastic completely. Due to slow degradation rate and increased utilization of plastic^5,12^, annually 25 million tons plastic waste gets accumulated in the environment^13–15^. Among the total accumulated plastic waste, polythene (PE) alone contributes about 64%^16^ and is considered as most problematic^17,18^. At dumping sites, terrestrial animals usually consume discarded plastic bags along with foodstuff and experiences severe health issues, which finally lead to their death^12,19^. All types of plastic wastes, finally enters into the marine environment through various routes and represents the maximum share (60–80%) of the marine waste by mass^20^. In the oceans, polythene waste emerged as a potential threat to the marine animals, leads to hamper their digestive tract and results in death of millions of marine animals^8,21–23^.

To minimize the production of plastic waste, different guidelines were adopted by various commissions and pollution control boards across the world. In India, Central Pollution Control Board working under the Ministry of Environment and Forests banned the production, dumping and marketing of the virgin/recycled carry bags with less than 20 micron thick^24^. Similarly Govt. of Maharashtra also banned the manufacturing and usage of the carrier bags below the thickness range of 50 micron^25^. Despite the ban imposed, various small grocery shops, fruit and vegetable stalls still uses these thin single use polythene bags of 20-micron thick illegally. Plastic wastes which forms an estimated quantity of 5–10% of total municipal solid waste, is being generated at the rate of about 1.2 lakh tons per day (TPD), of which 6000 TPD is plastic wastes^26^. The lack of provision for the proper disposal of post-consumer plastic wastes, results in littering on road which often chokes open drainage systems,  and leads to flood like condition during rainy seasons. At dumping sites, it mixes with the soil and release of the toxic compounds makes fertile land, infertile. So in order to tackle the menace of 20 micron thick plastic, it is necessary to find out way for its degradation. Despite being imposing restrictions on the usage of the plastic, still the plastic waste is generating at an alarming rate, so the disposal of the plastic waste emerged as a major challenge to deal with, throughout the world. Since, the discovery of the polythene, people tried to dispose polythene (plastic) waste using various strategies viz. landfilling (65%)^27–30^, incineration (25%)^27,31,32^, recycling (10%)^27,31,33^, producing biodegradable plastic^34–36^, construction of roads^37–39^, production of fuel^40–42^, degradation^3,12,43^, and biodegradation^44^. Each of the method is having, either deteriorating effects on the environment or economic exploitation and among all the methods, biodegradation is considered as the most accepted and ecofriendly method^3,12^. The degradation of the synthetic plastic mediated by the microbes is known as biodegradation^15,45,46^. Biodegradation of natural and synthetic plastics is carried out by microbes like Bacteria, Fungi and Actinomycetes^47^ under optimal growth conditions of the respective microbes in soil^10^. Rate of biodegradation is directly proportional to the molecular weight of the plastic targeted^8,47^, and can be enhanced by various factors viz. abiotic hydrolysis, photo–oxidation, and physical disintegration^10^. Various microorganism are reported to produce some special enzymes viz. intracellular and extracellular, which enabled the microbes to disintegrate the polymer into several monomers and dimers, which are being used by the microbes as a carbon source^48–50^ and results in the conversion of the polythene waste into water, CO_2_ or methane^3^. Efficiency of biodegradation can be increased by making polythene susceptible for microbial attack by using starch and pro-oxidant as additives of the plastic^51^. By the addition of starch during the preparation of polythene, hydrophilic nature of the polythene gets improved and enables some microbes to get attached on the surface of the polythene and results in de-polymerization with ease due to release of amylase enzyme^27^. As per Muthukumar and Veerappapilli^8^ growth of fungi can penetrate into the polymer and leads to its degradation. In past, polythene deteriorating fungi were reported from plastic waste dumping sites^52^, mangrove rhizosphere soil^4,53^ and marine water^45^.

So, in the current investigation, efforts were made to select those plastic waste dumping sites with growing mangroves surrounded by marine water along the West Coast of India for collection of the rhizosphere soil of *Avicennia marina* (Forsk.) Vierh., to isolate, screen, and characterize the potential polythene degrading fungi.

## Results

### Collection of the soil samples

**Figure 1:**
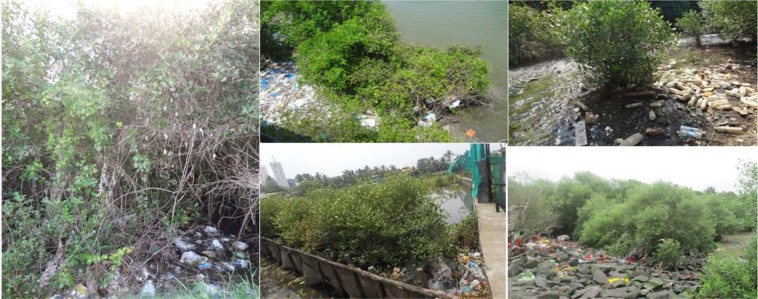
Representative collection sites of *A. marina* along the West Coast of India.

The rhizosphere soil samples of *Avicennia marina* (Forsk.) Vierh. (Fig. 1) were collected from 12 different eco-geographical locations along the West Coast of India (Supplementary Fig. S1). The longitude, latitude and altitude (from mean sea level) of each locality was also recorded (Supplementary Table S1).

### Isolation of Fungi

Total 109 fungal isolates were recorded from the collected rhizosphere soil. Sabouraud Dextrose Agar medium was found to be the best for the cultivation of the fungi (Supplementary Table S1). Maximum (31) fungal isolates were recorded from Mangalore locality whereas minimum (4) fungal isolates were reported in Surat, Mirya Bandar and Pudponnani.

### Investigation of polythene (PE) biodegradation using the fungal isolates

The potential polythene degrading fungi were screened on the basis of reduction in weight (percent weight loss: %WL) and tensile strength (percent loss in Tensile strength: % loss in TS) of the polythene after 60 days of incubation at ambient temperature with continuous shaking.

### Assessment of the PE deteriorating fungi based on percent reduction in weight

#### Screening of the PE degrading fungi at pH 3.5

After 60 days of incubation with continuous shaking at ambient temperature, among 109 fungal isolates, maximum percent reduction in weight or percent loss in weight (% WL) of the pretreated PE strips was recorded with BAYF6 (23.31 ± 1.88) at pH3.5 whereas least %WL was recorded with VASF8 (0.98 ± 0.02) (Supplementary Fig. S2). In addition to % WL, we also recorded weight gain with various isolates. Maximum percent weight gain (%WG) of the polythene was recorded with PODPF2 (13.37 ± 4.72).

#### Screening of PE degrading fungi at pH 7

Among the 109 fungal isolates maximum % WL was recorded with MANGF1 (58.51 ± 8.14) followed by ERNF1 with 37.94 ± 3.06%WL whereas minimum % WL (1.38 ± 0.54) was recorded with SURF3 at pH 7 (Supplementary Fig. S3) after 60 days of continuous shaking at ambient temperature. Similar to pH 3.5 percent weight gain (%WG) was also recorded at pH 7. Maximum weight gain (7.43 ± 1.98) was recorded with OLDGF2.

#### Screening of PE degrading fungi at pH 9.5

Maximum % WL (41.82 ± 5.47) was recorded with MANGF1, among the total 109 fungal isolates after 2 months of regular shaking at room temperature at pH 9.5 (Supplementary Fig. S4). With various isolates no loss or gain of the percent weight was recorded. Least % WL (0.64 ± 0.22) was recorded with SURF3. Similar to pH 3.5 and pH7, weight gain was also reported at pH 9.5. At pH 9.5 highest percent weight gain (28.41 ± 6.99) was recorded with MANGF13 compared to pH 3.5 and pH 7.

Among the 109 fungi, maximum %WL (58.51 ± 8.14) was recorded at pH7 with BAYF5 (Supplementary Table S2) followed by MANGF1 (41.82 ± 5.47) at pH 9.5.

#### Reproducibility of the results based on %WL

Reproducibility of the data is the most important component of a successful experiment. To validate the replicability the results, three most efficient polythene degrading fungi were used for repetition experiment. It was noticed that the results based on the repetition experiment were different than the previous results. In screening BAYF5 leads maximum % WL at pH 7 but in repetition experiment we observed maximum reduction in weight (≈50%WL) with MANGF1 (Supplementary Fig. S5).

### Assessment of the PE deteriorating fungi based on reduction in tensile strength (TS)

#### Screening of PE degrading fungi at pH 3.5

Among the 109 fungi, maximum percent reduction in TS or % loss in TS (94.44 ± 2.41) was reported with PNPF15 at pH 3.5 (Supplementary Fig. 6) and the least % loss in TS (2.5 ± 0.42) was reported with the isolate BAYF8 respectively.

#### Screening of PE degrading fungi at pH 7

Based on % loss in TS, the most efficient polythene degrading fungal isolate at pH 7 was VASF1 with potential of 76.04 ± 5.21% loss in TS in 60 days of incubation period (Supplementary Fig. S7). Fungal isolate JAMNF5 was found to have least activity and leads only 1.67 ± 0.42% loss in TS in the same period.

#### Screening of PE degrading fungi at pH 9.5

At pH 9.5 the fungal isolate VASF6 recorded 62.50 ± 4.17% loss in TS of polythene which is the maximum among the total fungi (Supplementary Fig. S8) whereas the least % loss in TS (1.25 ± 0.42) was documented with BAYF6 at the same pH after 60 days of incubation period.

Among the 109 fungal isolates at three different pH, the top 5 elite potential polythene deteriorating fungi based % loss in TS are enlisted in Supplementary Table S3.

#### Reproducibility of the results based on percent reduction in TS

During the repetition experiment the results were found similar to that of screening (Supplementary Fig. 9) and proved reproducible compared to the %WL after 60 days of continuous shaking at ambient temperature.

### Confirmation of PE degradation with fungi

#### Scanning electron microscopic (SEM) analysis

**Figure 2:**
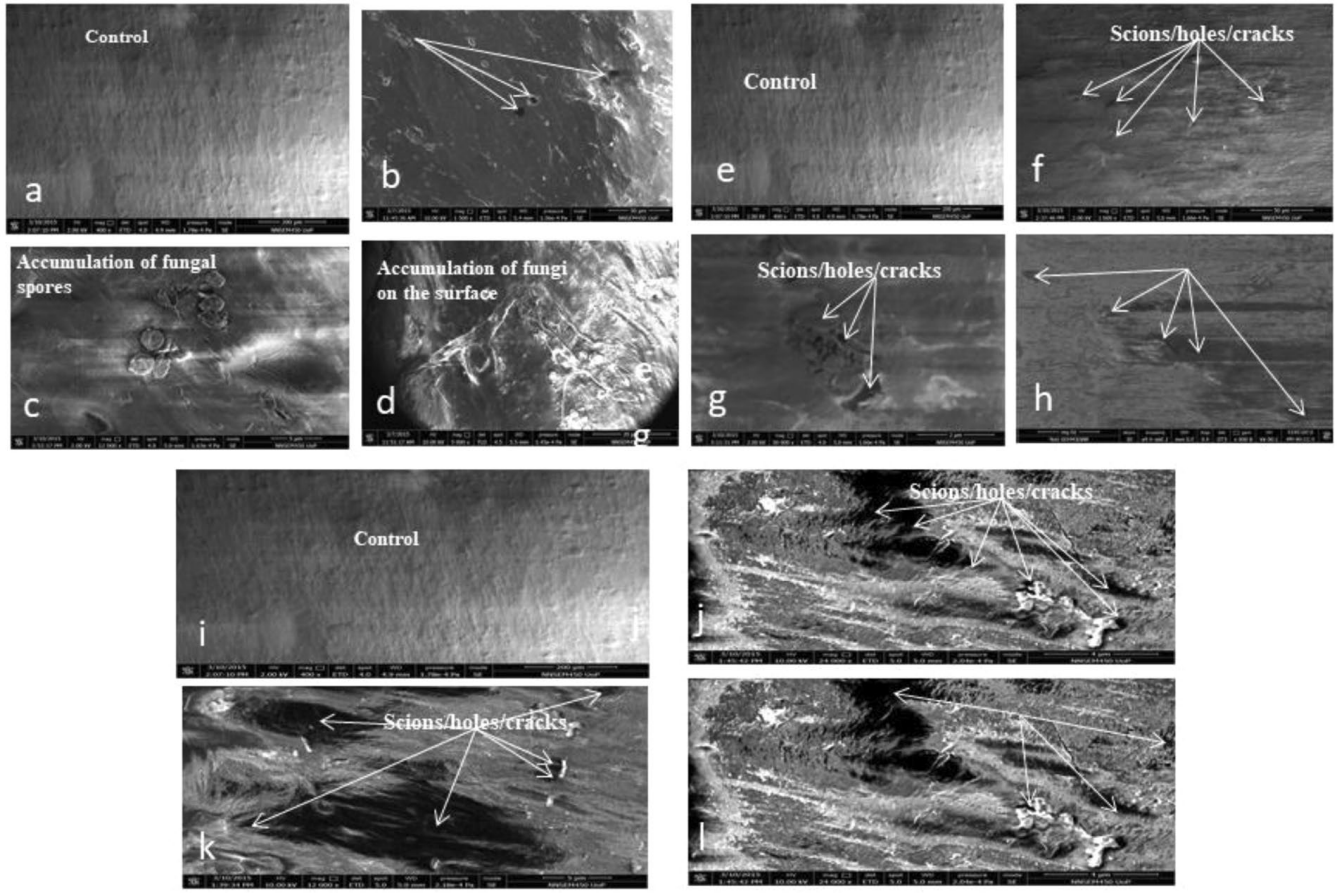
Scanning Electron Microscopic image of the polythene strips: (**a**–**d**) SEM of the PE with most %weight gain (24.4%) by JAMNF at pH9.5; (**e**–**h**) SEM of PE strip with maximum (94) % loss in TS by PNPF-15 at pH 3.5; (**i**–**l**) SEM of PE strips of maximum %WL (41%) by MANGF1.

The results of degradation of the polythene strips by the fungal isolates were confirmed by the formation of the cracks/holes/scions and were visualized in Scanning electron microscopic photographs (Fig. 2).

#### Fourier-transform infrared spectroscopy (FTIR) analysis

**Figure 3:**
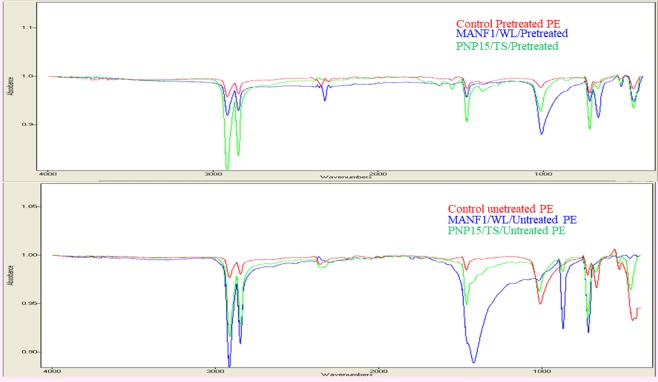
FTIR spectra: A. pretreated PE strip. a: control, b: MANGF1/WL, c: PNP15/TS. B. untreated PE strip. a: control, b: MANGF1/WL, c: PNP15/TS.

The degradation of the polythene was also confirmed with FTIR analysis (Fig. 3; Supplementary Table S4) in terms of changes in Carbonyl Index. Maximum change in Carbonyl Index was recorded in untreated polythene (4.36 and 4.34) with both the fungi (MANGF1/WL and PNP15/TS respectively) compared to pre-treated PE strips (2.56 and 2.63).

### Characterization of the most efficient polythene degrading fungal Isolates

#### Identification of fungi based at morphological keys

Based on the morphological keys, the fungal isolates were identified up to genus level. Among the ten elite polythene degrading fungi, 8 isolates (BAYF5/WL; MANGF1/WL; ERNF1/WL; BAYF7/WL; MANGF2/WL; PNPF15/TS; VASF1/TS and VASF6/TS) were reported to belong the genus *Aspergillus*, whereas, one isolate (ERNF3/WL) was not identified at morphological level and the isolate MIRF3/TS represent the genus *Penicillium* sp. (Supplementary Fig. S10; Supplementary Fig. S11).

#### Molecular characterization of the potential PE degrading fungi

**Figure 4:**
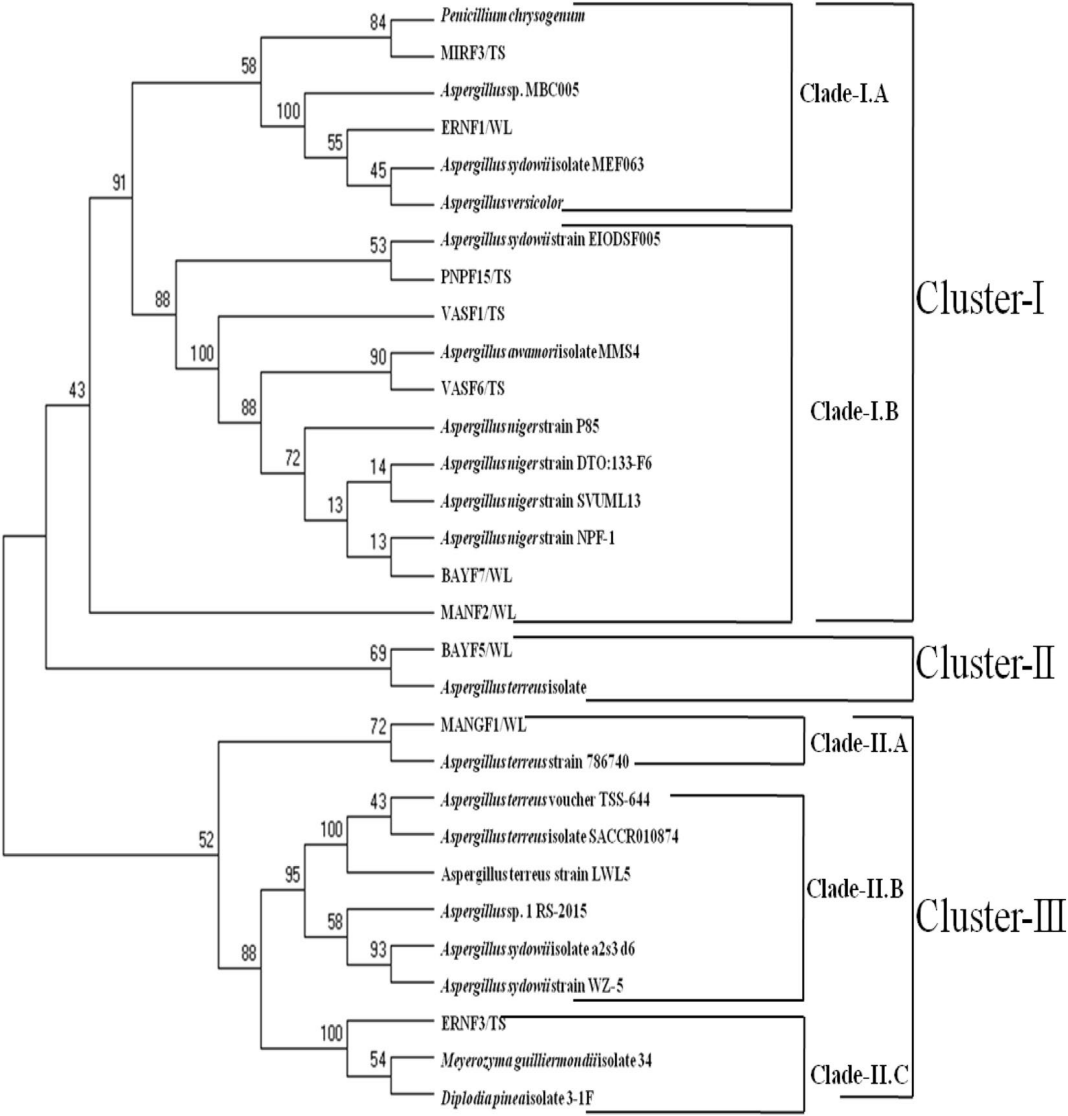
Molecular phylogenetic analysis of polythene degrading fungi by maximum likelihood method along with the homologous ITS sequences retrieved from the gene bank (NCBI).

The amplified ITS genes of the polythene degrading fungi were separated on 1.2% Agarose gel against 100 base DNA ladder along with the negative control (Supplementary Fig. S12). All the sequences were accessioned by the gene bank (NCBI) (Supplementary Table S5). The polythene degrading fungal isolates were characterized based on sequence homology of internal transcribed spacer (ITS) gene. All the ITS sequences of the top 10 fungal isolates along with the ITS gene homology sequences retrieved from the gene bank were clustered into three main groups (Fig. 4). Group one is the largest cluster and further sub-clustered into two clades (clade I.A and clade I.B). In clade-I.A only two fungal isolates, MIRF3/TS and ERNF1/TS were grouped with other homologous sequences obtained from the gene bank and were identified as *Penicillium chrysogenum* strain MIRF3/TS and *Aspergillus sydowii* strain ERNF1/TS. Four fungal isolates, BAYF7/WL, VASF6/TS, VASF1/TS and PNPF15/TS were grouped with clade I.B and were identified as *Aspergillus niger* strain BAYF7/WL, *Aspergillus awamori* strain VASF6/TS, *Aspergillus awamori* strain VASF1/TS and *Aspergillus sydowii* strain PNPF15/TS. In cluster II only BAYF5/WL was grouped, and was identified as *Aspergillus terreus* strain BAYF5/WL. Cluster-III was grouped into three main clades (clade-II.A, clade-II.B and clade-II.C). In clade-II.A only one fungal isolate was clustered and identified as *Aspergillus terreus* strain MANGF1/WL. Similar to clade-II.A, only one fungal isolate was grouped with clade-II.C and was identified as *Meyerozyma guilliermondii* strain ERNF3/TS.

## Discussion

Polythene waste alone shares 64% of the total plastic waste produced annually across the globe^12,16^. Among all the methods available to deal with plastic waste disposal, bioremediation technology succeeded with wide range of acceptance throughout the globe^3,12^. In literature, polythene deteriorating fungi were reported from various sources viz. marine water, plastic dumping sites and mangrove rhizosphere soil^3,12,16^. In the current investigation, we selected only those sites, for collecting of the rhizosphere soil samples to isolate polythene degrading fungi which represent all these sources. There are two reports^4,54^ from East Coast of India and one report^4^ from South East Coast of India and each report exhibits either utilization of mangrove rhizosphere soil or marine water for isolation of polythene degrading fungi. To the best of our knowledge, from the West coast of India, we have reported for the first time, polythene degrading fungi from all the available polythene degrading sources (dumping site, mangrove rhizosphere, marine water). Polythene degradation using fungal isolates had been assessed by determining the changes in some of the key characteristics of the polythene before and after the treatment of the fungal isolates viz. reduction in weight, reduction in tensile strength, reduction in percent elongation, reduction in viscosity, reduction in crystallinity, formation of cracks/scars/holes on the surface of the polythene, changes in the carbonyl index, estimation of CO_2_ released etc^3,12^. The rate of polythene degradation is highly influenced with various other factors viz. incubation time, pH, temperature, treatment of the polythene with some acids e.g. nitric acid (to remove the plasticizers) etc^3,12^. The incubation time period (period from the date of treatment to harvesting of the polythene strips for assessing the level of polythene deterioration) of the fungal isolates used to determine the rate of polythene degradation reports to be varied from 10 days to 32 years.

Previously after 30 days of incubation maximum 28.80 ± 2.40 percent weight loss (%WL) of the polythene was recorded with *Aspergillus glaucus*^4^, 23.11% WL (pre-treated UV and nitric acid) with *Aspergillus niger*^55^, 12.25%WL with *Aspergillus niger*^56^, 11.11% WL (LDPE) with *Aspergillus japonicas* and 5.8% with *Aspergillus niger*^57^. Even after increasing the incubation duration to 60 days, only 28–40% WL the polythene with *Aspergillus niger* was recorded but our results are more promising and efficient comparatively, we have reported ≈50% WL of the pretreated polythene strips with *Aspergillus terreus* MANGF1/WL at pH 9.5 during the same incubation period (60 days). Various workers used 3 months incubation time to determine the level of polythene degradation using fungi and recorded maximum 58.45% WL in pretreated polythene (2 days old chemically treated polythene followed by UV irradiation for 50 minutes before treatment) with *Aspergillus oryzae*, whereas in case of untreated polythene, only 6.3%WL with the same fungi during the same test period was recorded^58^ followed by 5.95% WL (with *Aspergillus niger)*^59^, 1.2% WL (with *Curvularia lunata)*, 0.8% WL (with *Alternaria alternate)*, 7.7% WL (*Penicellium simplicissimum)* and 0.7% WL (with *Fusarium* sp.) but the consortia of all these fungi (*Curvularia lunata* + *Alternaria alternate* + *Penicellium simplicissimum* + *Fusarium* sp.) results in 27% WL during the same test period^60^. After 6 months of incubation, only 26.17% WL (with *Aspergillus niger*)^61^ and 20.63%WL (with *Aspergillus flavus*)^62^ of polythene was documented. Aswale^63^ reported maximum 50%WL (with *Phanerochaete* *chrysososporium*) of the polythene at pH 4 after 8 months of incubation period. Abdullahi *et al*.^64^ recorded polythene degradation after 9 months of test period in terms of percent weight loss in two different types of degradation sets (polythene seeding in cow dung mixed fadama soil (PECDS) and poultry dropping mixed fadama soil (PEPDS) and reported 18.1% WL in the set of PECDS whereas only 6.0% WL with set PEPDS. Finally they concluded *Aspergillus niger*, *A. fumigatues*, and *A. flavus* mixed with PECDS and PEPDS leads to highest weight loss of the polythene compared to fadama soil mixed with inorganic fertilizer (NPK) and control. The possible reason for weight reduction in polythene in all the studies is due to breakdown of carbon backbone (enzymatic degradation)^65^ and utilizing the resulting monomers and oligomers as a carbon source by the fungi^61,66^. Otake *et al*.^67^ buried different kinds of plastic (including polythene) in the soil and assessed the level of degradation after 32 years and reported only whitening patches on the surface of the polythene due to the microbes (both fungi and bacteria) and recorded no evidence of degradation of other types of plastics. Among the various reports^68–72^ published during 2017–2018 on fungal based plastic degradation, *Penicillium* sp. was recorded as the most efficient fungi with percent weight reduction 43.4%^70^ in just 30 days.

Besides reduction in weight due to the degradation potential of the fungi, reduction of the tensile strength is also one of the widely studied parameter by different research groups around the globe. In the current investigation, we reported highest percent reduction in TS (94.44 ± 2.40%) with *A. sydowii* strain PNPF15/TS at pH 3.5 after 60 days of continuous shaking at ambient temperature. Our results are in agreement with the previous studies, previously, after 10 days of incubation maximum reduction (60%) in tensile strength (TS) of the heat treated polythene was reported with *Mucor rouxii* [NRRL 1835]^73^. After three months of testing period maximum reduction in TS (63%) of the polythene was reported with *A. oryzae*^58^ followed by 51% reduction in PE (Mangnease sterate treated LDPE exposed to UV irradiation) with the same fungi (*A. oryzae*)^74^. Vijaya and Reddy^52^ followed the ASTM standard and assessed the degradation (by compositing) of polythene (HDPE) along with municipality solid waste and recorded highest 20% reduction in tensile of HDPE after 1 year of testing duration. Vijaya and Reddy^52^ studied correlation coefficient among WL and TS and suggested strong correlation coefficient; if one factor is affected by microbial attack other factor also  gets affected at the same time.

The degradation of the polythene was further authenticated using SEM and FTIR analysis. The SEM analysis revealed the degradation level on the surface of the polythene in the form craks/scions/holes (Fig. 2). Our observations are similar with the previous reports. Due to use of SEM analysis, structural changes and erosions on the surface of the polythene in the form of porosity, cavities, holes/scions/cracks were reported with fungal consortia^60^, *Mucor circinilloides* and *Aspergillus flavus*^45^, *Aspergillus* and *Penicillium*^75^, *Chaetomium globosum*^76^, *Aspergillus niger* and *Aspergillus japonicas*^57,77^. After SEM analysis, the level of polythene degradation was further authenticated by FTIR analysis. In the present investigation, the FTIR data confirmed the level of structural changes in the polythene. Abiotically treated sample (HNO_3_ treatment, 20 min UV treatment) shows generation of carbonyl peak, carboxylic acid and its derivatives. We observed the peak of carboxylic acid in the range of 1633.73–1812.08 cm^−1^ and reported the reduction of this peak up to 1629.53 cm^−1^ and no peak was observed at 1812.08 cm^−1^ on the PE strips degraded by *A. terreus* strain MANGF1/WL. *A. sydowii* strain PNPF15/TS based degraded polythene strip depicts the reduction of peak from 1633.73–1812.08 cm^−1^ to 1628.60 cm^−1^ and similarly no peak was recorded at 1812.08. Similarly in past, Konduri *et al*.^74^ also reported carboxylic acid peak in the range of 1630–1840 cm^−1^.

Chatterjee *et al*.^78^ reported the formation of C-H stress group at peak 2915 and we also reported similar peak at 2912.10 cm^−1^ in abiotic treated PE (control) and documented the reduction in the peak with both the fungal strains (*A. terreus* strain MANGF1/WL and *A. sydowii* strain PNPF15/TS) to 2912.09 cm^−1^ and 2912.92 cm^−1^ respectively. Chatterjee *et al*.^78^ reported occurrence of CH_2_ peak at 718, same functional group was observed in our study in the control PE strip at peak 721.52 cm^−1^ and compared to control we reported reduction in the peak with *A. terreus* strain MANGF1/WL (720.88 cm^−1^) and *A. sydowii* strain PNPF15/TS (721.05 cm^−1^). Balasubramanian *et al*.^79^ reported Keto carbonyl band at 1715, we reported the similar peak (1716.66 cm^−1^) in the control PE strips, but due to action of both the fungal strains this Keto carbonyl peak was not recorded on the degraded PE strips. Konduri *et al*.^74^ reported the peak of C=O stretching in between 1710–1740 cm^−1^, we got nearly same but smallar peak in between 1716–1766 cm^−1^ in the control PE strips and similar to Keto carbonyl band, C=O stretching was also not observed in degraded PE strips with both the fungal strains. In case of untreated PE strips (control), carboxylic group peak was not recorded, whereas CH_2_ was recorded at peak 721.28 cm^−1^ and reduction in CH_2_ peak was reported in the untreated PE strips degraded by both the fungi (718.45 cm^−1^ by *A. terreus* strain MANGF1/WL and 720.51 cm^−1^ by *A. sydowii* strain PNPF15/TS). CH stress peak was observed in the control PE (untreated) strips at 2913.03 cm^−1^ and only *A. sydowii* strain PNPF15/TS was reported to lead reduction of CH peak to 2912.02. In agreement with the Konduri *et al*.^74^ we also recorded C=C stretching at two peaks 1739.35 cm^−1^ and 1792.57 cm^-1^ only in case of PE strips degraded by *A. terreus* strain MANGF1/WL. Microbes are also reported to be responsible for decreasing the carbonyl index^58^ which in turn depicts the level of degradation. Manzur *et al*.^80^ reported maximum (40%) reduction in Carbonyl Index (CI) after 3 months of incubation. Yamada *et al*.^81^ studied the effect of *Penicillium simplicissimum* (soil fungi) on the degradation of low density polythene and after 3 months of incubation in liquid culture, *Penicillium simplicissimum* was reported to utilize polythene as a carbon source before irradiating with UV and nitric acid treatment. They also suggested that time required for degradation of polythene is depend on the time needed for the growth phase pure culture and they also reported that degradation is directly proportional to the addition of functional groups. Konduri *et al*.^58,74^ observed reduction in carbonyl group after three and six months of incubation with the fungi *A. oryzae* and *A. flavus*. Similarly in the current investigation there was a change in carbonyl groups, carboxylic groups after incubation with *A. terreus* strain MANGF/WL and *A. sydowii* strain PNPF15/TS for 60 days of continuous shaking at ambient temperature.

The previous literature depicts that polythene deteriorating fungi were mostly characterized based on morphological keys^82–84^. There are only few reports of identification of polythene degrading fungi at biochemical level^82,85^ and at molecular level^5,58,69^. As per the literature the traditional methods of fungal identification are time consuming, labour extensive and needs the utility of wide range of culture media with experienced personnel to characterize commonly occurring fungal strain variants^86–88^. The traditional methods are usually based on morphological keys and biochemical tests such as the identification of yeast based on biochemical test such as carbohydrate assimilation and fermentation tests which are unmanageable in non-specialized laboratory of microbiology^89^. There are various kits available in the market which leads to the rapid identification of the fungi but these kits are also not reliable and may takes few weeks to get the final results^90,91^. So, it is needed to have fast and accurate method of fungi identification. In the present scenario, the strategy utilized to identify many important fungi is the combined usage of morphological keys and biochemical tests with molecular diagnostics^92^. Presently, molecular tools are employed to aid the traditional method of fungi identification at greater pace^93–95^. Analysis of the variation in the internal transcribed spacer (ITS) regions of the rDNA is widely used for accurate identification of fungi^96^. Identification species and strain are more accurate based on variation in the ITSl/ITS2 domains than the 18S region (small subunit), the 5.8S region and the 28S region (large subunit)^96^^,^^97^. As per reports^98^^,^^99^ sequence based method is most rapid and authentic. Furthermore, molecular tools are the authentic and more reliable than the morphological and biochemical analysis and are considered as gold standard for the identification of any micro-organism. In the current investigation, based on morphological and molecular level (ITS gene sequence variation analysis), *Aspergillus*, *Penicillium* and *Meyerozyma* were reported as three main polythene degrading fungal genera. In case of genus *Aspergillus*, only four species in agreement with the previous reports such as *Aspergillus awamori*^100^; *A. niger*^52,83,101^, *A. terreus*^57,102^ and *A. versicolor*^102^ were recorded with polythene degradation potential. Besides the above species of the *Aspergillus* 7 more species such as *A. candidus*^52^, *A. cremeus*^52^, A. glaucus^4,101^, *A. japonicus*^57^, *A. nidulans*^82^^,^^62^,  *A. flavus*^52,82^, *A. oryzae*^103^, *A. ornatus*^52^ were reported to have polythene degradation potential. The fungus, *Aspergillus sydowii* from the genus *Aspergillus* was reported for the first time with the polythene degradation potential, however, it was reported to degrade PVC plastic^5^. In genus *Penicillium* only *Penicillium chrysogenum* was recorded in the current investigation to degrade polythene. Sowmya *et al*.^60^ studied the degradation of rubber due to *Penicillium chrysogenum*, however, in literature *Penicillium simlicimmum*^81^, *Penicillium*sp.^82^, *P*. *pinophilum*^83^, *P. frequentans*^82^, *P. funiculosum*^104^, also reported to have polythene degradation capacity. In the literature there is no report of *Meyerozyma guilliermondii* with polythene deteriorating potential, instead gasoline was reported to be degraded with same fungi^105^. Further, the polythene degradation-products (PE-DP) produced with the elite polythene degrading fungi (*Aspergillus terreus* strain MANGF1/WL and *Aspergillus sydowii* strain PNPF15/TS) were subjected to Gas Chromatography and Mass Spectra analysis followed by followed by their deleterious potential effect on Sorghum seeds and tiger shark fish were assessed, fungi based by-products of the polythene were found least toxic to both the plants and animal system^106^.

## Methods

### Collection and transportation of mangrove rhizosphere soil

Soil samples were collected from rhizosphere of *A. marina* from 12 different locations along the West Coast of India through individual and group visits (Supplementary Fig. S1 and Supplementary Table S1) and were transported to the laboratory as per standard method stated in our previous study^107^.

### Isolation of the fungi using serial dilution

From the collected rhizosphere soil samples, fungal isolates were obtained by following serial dilution method, the fungal isolates were grown on Sabouraud’s Agar media (SA media), and the axenic cultures were also maintained as per the standard protocols^108^.

### Screening of the polythene degrading fungi

From each fungal isolate (from six days old pure culture grown on Sabouraud’s broth) 1 ml (fungal) culture was used as an inoculum (average inoculum size 9.52 × 10^2^ CFU) were screened based on their potential to degrade polythene at varied pH with regular shaking at room temperature. After 60 days of continuous shaking, polythene degradation was assessed using percent reduction or loss in weight (%WL) and percent reduction or loss in tensile strength (% loss in TS).

#### Reproducibility of the fungal based polythene degradation results

After screening, most efficient polythene degrading fungi based on percent reduction or loss in weight (Top 2 fungal isolates) and reduction or loss in tensile strength (Top 2 fungal isolates) along with control, were subjected to repetition under the same conditions as applied during screening, to infer the reproducibility of the polythene degradation results. At this stage, two types of polythene strips viz. pre-treated and untreated strips were used.

### Confirmation of the polythene degrading potential of the fungi

After screening, the most efficient polythene degrading fungi were subjected to Scanning Electron Microscopy analysis and Fourier-transform infrared spectroscopy (FTIR) analysis to confirm the level of polythene degradation as per the methods stated in our previous study^107^.

### Characterization of the most efficient polythene degrading fungal isolates

The most efficient fungal isolates with potential to degrade polythene (5 based on %WL and 5 based on % loss in TS) were characterized based on morphological keys^109^ and molecular tools. At morphological level, microphotographs of the selected fungal isolates were captured using trinocular microscope (Leica DM3000, Germany) equipped with cooled CCD camera (Leica DFC450, Germany). The captured photographs were processed by Leica Application Suite (Version 4.5.0). Cetyl Trimethyl Ammonium Bromide (CTAB) method^110^ was used to isolate genomic DNA from the 6 days old fungal cultures (100 mg of fungal mycelium) to identify the elite polythene degrading fungi at molecular level. The PCR reaction (25 μl) was carried out using 1.5 mM MgCl_2_, 0.25 mM of each dNTPs, 1X *Taq* buffer, 1 U/μl *Taq* DNA polymerase, 10 pmole of each primer of ITS gene^93^ (Supplementary Table S6) and 50 ng DNA template. The PCR (Veriti, gradient thermocyler cycler, Applied Biosystem, USA) was programmed at initial denaturation 95 °C 5 min, 40 cycles with denaturation at 95 °C 1 min, annealing 59 °C 1 min, extension 72 °C 1 min followed by final extension at 72 °C 10 min. The PCR products were separated on 1.2% Agarose gel (Invitrogen) prepared using 1X TAE buffer against negative control and 100 bp DNA ladder (Invitrogen). All the amplified bands were eluted from the Agarose gel using Qiagen gel extraction kit (Cat. No. 28115) as per the instruction manual and were given to commercial lab along with primers for purification and sequencing. The sequences obtained from the commercial lab were viewed using chromas lite and were curated to make contig using MEGA 6 software. The evolutionary history was inferred by using the Maximum Likelihood method based on the Tamura 3-parameter model^111^. The bootstrap consensus tree inferred from 1000 replicates^111^ was taken to represent the evolutionary history of the taxa analyzed^111^. Branches corresponding to partitions reproduced in less than 100% bootstrap replicates were collapsed. The percentage of replicate trees in which the associated taxa clustered together in the bootstrap test (1000 replicates) was shown above the branches^111^. Initial tree(s) for the heuristic search were obtained by applying the Neighbor-Joining method to a matrix of pair wise distances estimated using the Maximum Composite Likelihood (MCL) approach. The analysis involved 30 nucleotide sequences. There were a total of 1088 positions in the final dataset. Evolutionary analyses were conducted in MEGA6^112^.

All the ITS gene sequences of the top 10 polythene degrading fungi were submitted to gene bank (NCBI) and were accessioned (KU551273–KU551282). The pure fungal cultures of two most efficient polythene deteriorating fungal isolates in slants (duplicate) were submitted at Col. Sir R. N. Chopra, Microbial Resource Center Jammu (MRCJ), CSIR-Indian Institute of Integrative Medicine, Jammu, India for general deposition and were also accessioned (MRCJ-791 and MRCJ-792).

## Conclusions

Among the 109 fungal isolates, *Aspergillus terreus* strain MANGF1/WL (more than 50.00 ± 4% WL, pH 9.5) and *Aspergillus sydowii* strain PNPF15/TS (94.44 ± 2.40% loss in TS, pH 3.5) are the most efficient and elite polythene deteriorating fungi based on reduction in weight, reduction in tensile strength, SEM and FTIR analysis. SEM analysis of the surface of the degraded polythene showed disturbances such as cracks, scions, fissures and holes which confirms corrosion. FTIR analysis shows formation of carbonyl group (1710–1740 cm^−1^), carboxylic group (1630–1840 cm^−1^), CH stress (2915 cm^−1^) and CH_2_ group (720 cm^−1^) after the UV and chemical treatment in control. These peaks were found to be reduced after fungal treatment. These decreasing peaks are due to the consumption of carbonyl and carboxylic acid derivatives by fungi indicating the de-polymerization of the polythene chain.

## Supplementary information

Supplementary Information for Potential of fungi isolated from the dumping sites mangrove rhizosphere soil to degrade polythene

## Data Availability

Data would be available on request to corresponding author.
