# Supplementary material for: Potential of fungi isolated from the dumping sites mangrove rhizosphere soil to degrade polythene
Source: Sci Rep. 2019 Mar 29;9:5390. doi: 10.1038/s41598-019-41448-y (PMC6440974; doi:10.1038/s41598-019-41448-y)
Supplement: Supplementary file 1 — Supplementary Information for Potential of fungi isolated from the dumping sites mangrove rhizosphere soil to degrade polythene [file 41598_2019_41448_MOESM1_ESM.pdf]

Supplementary Information for

Potential of fungi isolated from the dumping sites mangrove rhizosphere soil to degrade polythene

Manisha K. Sangale#[1,2], Mohd. Shahnawaz#* [1,3], and Avinash B. Ade*[1]
[1]Department of Botany, Savitribai Phule Pune University, Pune-411007, Maharashtra, India.
[2] Present address, Department of Botany, S M Joshi College Hadapsar, Malwadi, Hadapsar, Pune, Maharashtra 411028, India
[3]Present address, Plant Biotechnology Division, CSIR-Indian Institute of Integrative Medicine, Canal Road Jammu, Jammu-180001, Jammu and Kashmir, India
*Corresponding author: Phone: +91-020-25601439. Fax: +91-020-25690498.
Email: mskhakii@unipune.ac.in
avinashade@unipune.ac.in
#authors contributed equally

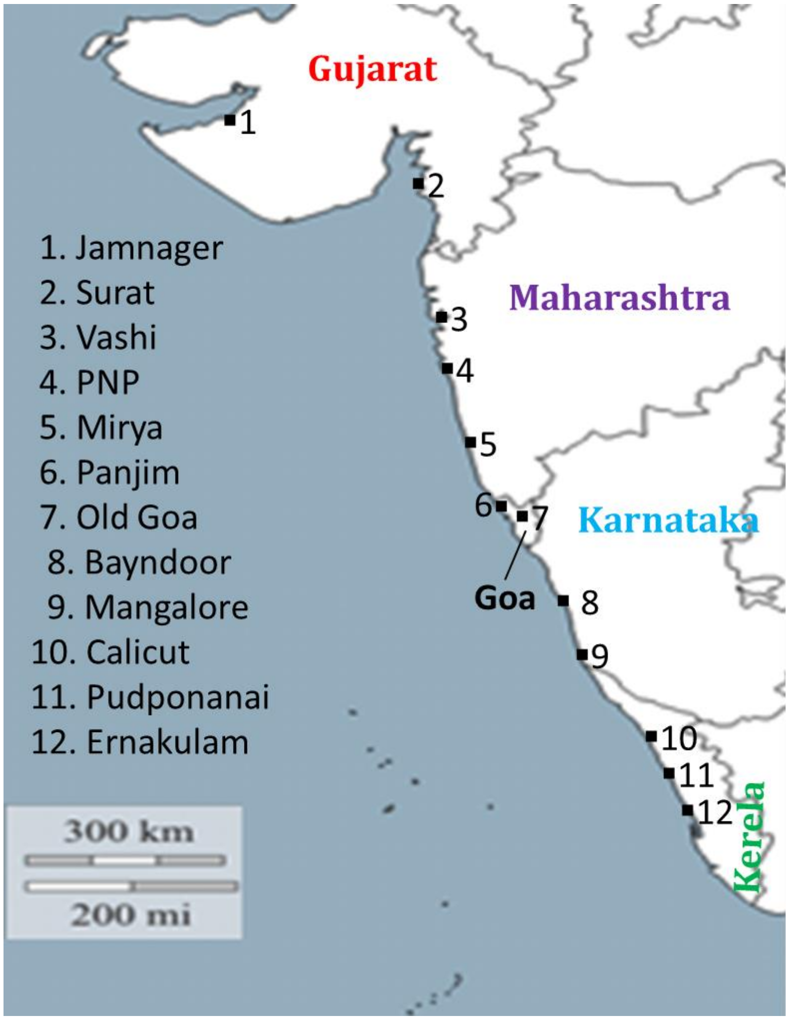

Supplementary Figure S1

Location of the rhizosphere soil collection sites of the of *A. marina* along the West Coast of India (Reprinted by permission from [Springer Nature]: [Environmental Science and Pollution Research] [Rhizosphere of *Avicennia marina* (Forsk.) Vierh. as a landmark for polythene degrading bacteria, Mohd. Shahnawaz, Manisha K. Sangale, Avinash B. Ade © 2016)

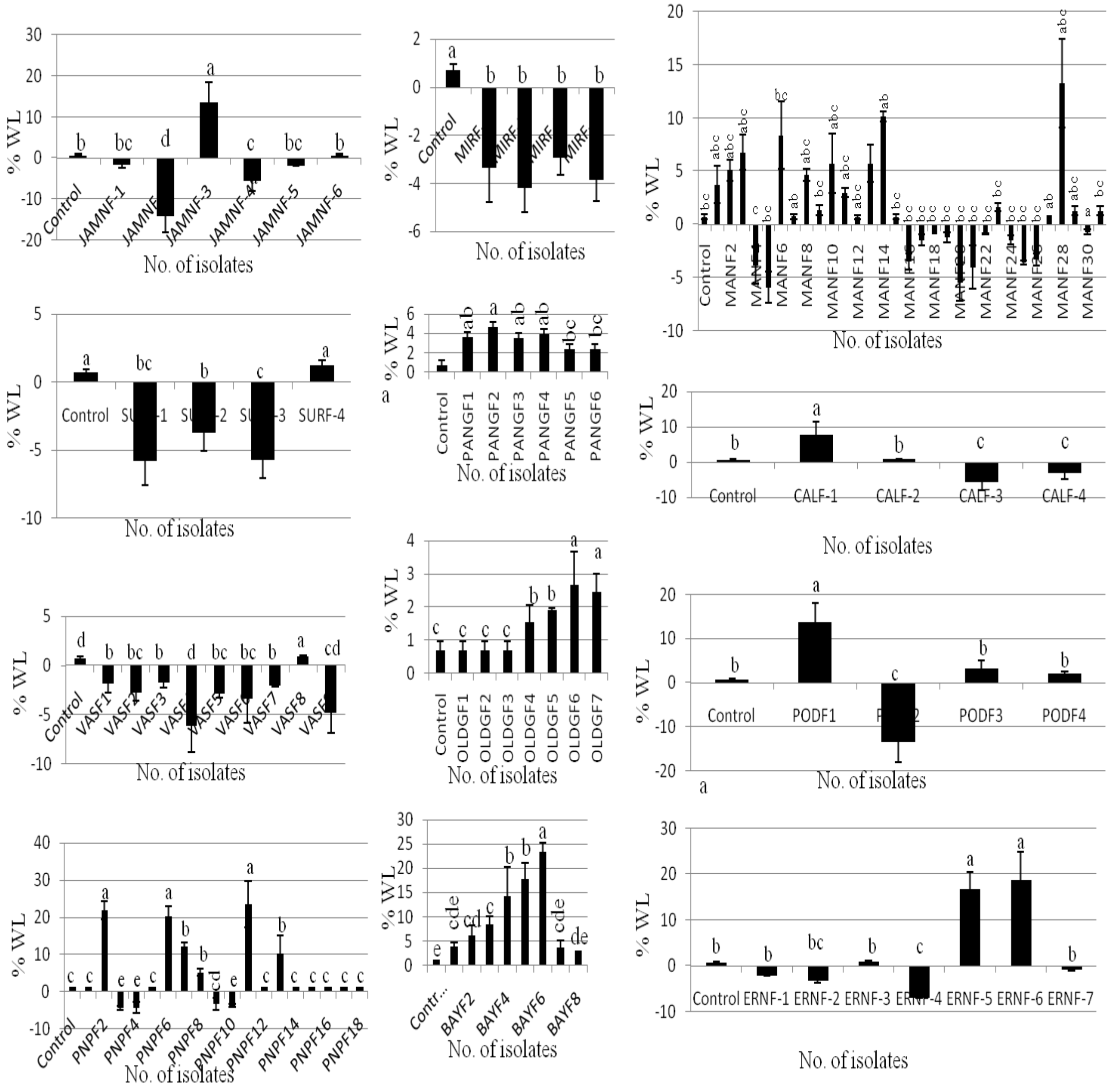

Supplementary Figure S2

Percent weight loss of PE at pH 3.5 (different lower case letters above the Standard deviation bars are significantly different at 0.05 level of significance).

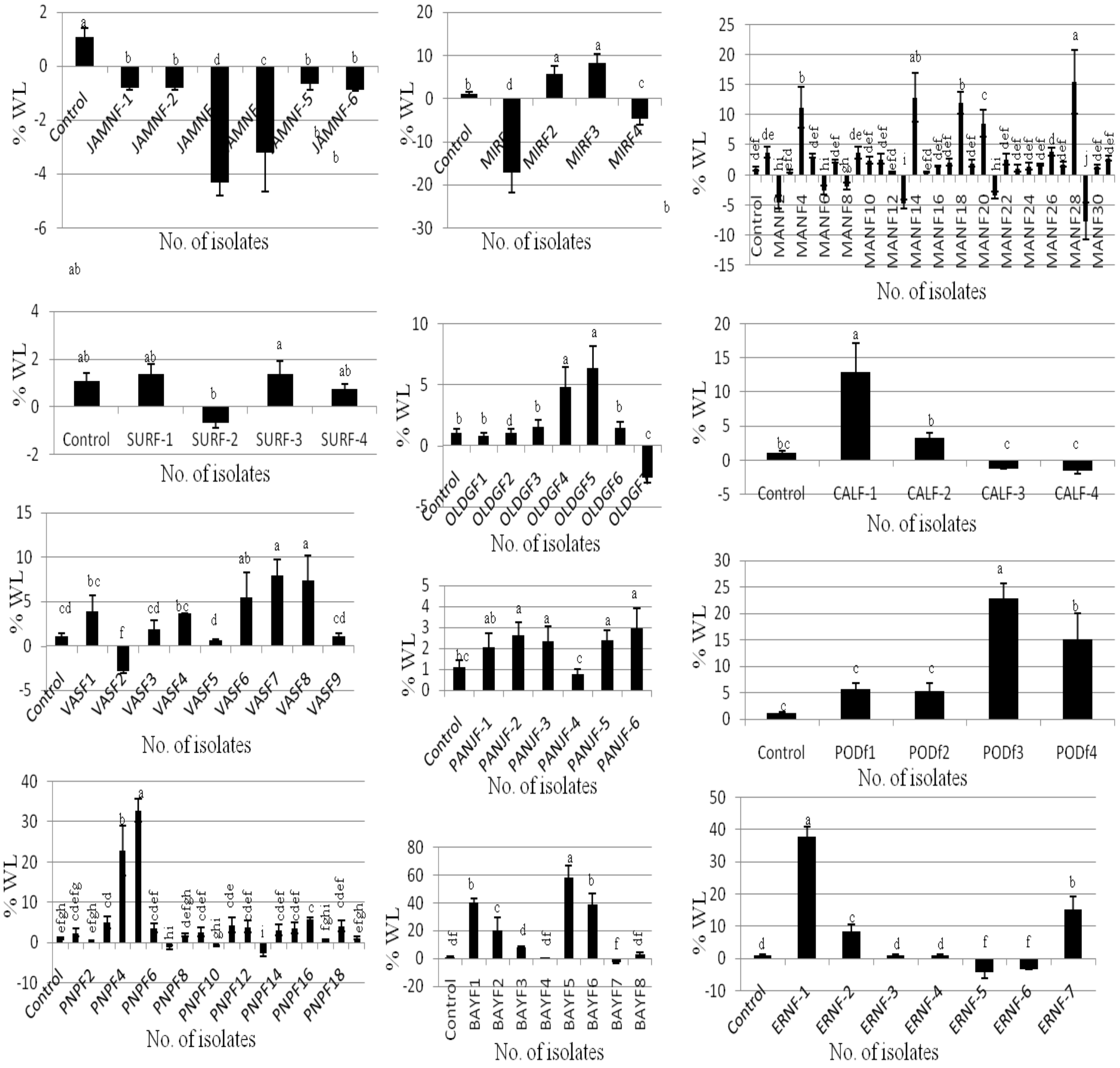

Supplementary Figure S3

Percent weight loss of PE at pH 7 (different lower case letters above the Standard deviation bars are significantly different at 0.05 level of significance).

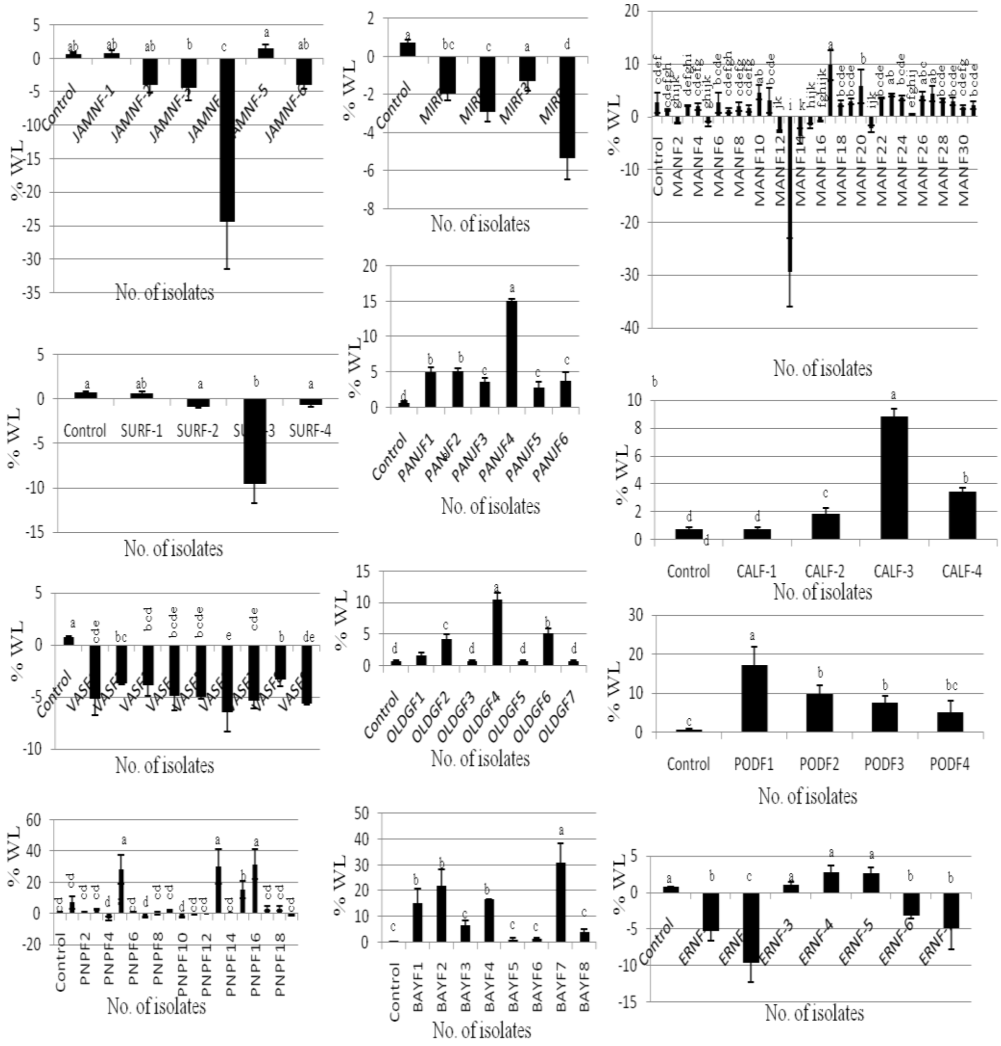

Supplementary Figure S4

Percent weight loss of PE at pH 9.5 (different lower case letters above the standard deviation bars are significantly different at 0.05 level of significance).

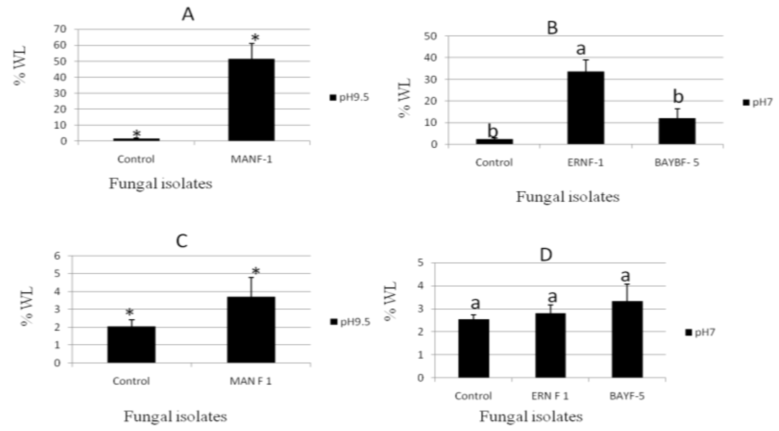

Supplementary Figure S5
Repetition based on % WL: A-B: repetition based on % WL in pretreated PE; C- D: repetition based on % WL in untreated PE (different lower case letters above the standard deviation bars are significantly different at 0.05 level of significance based on ANOVA; *statistically different based on t-test at 0.05 level of significance).

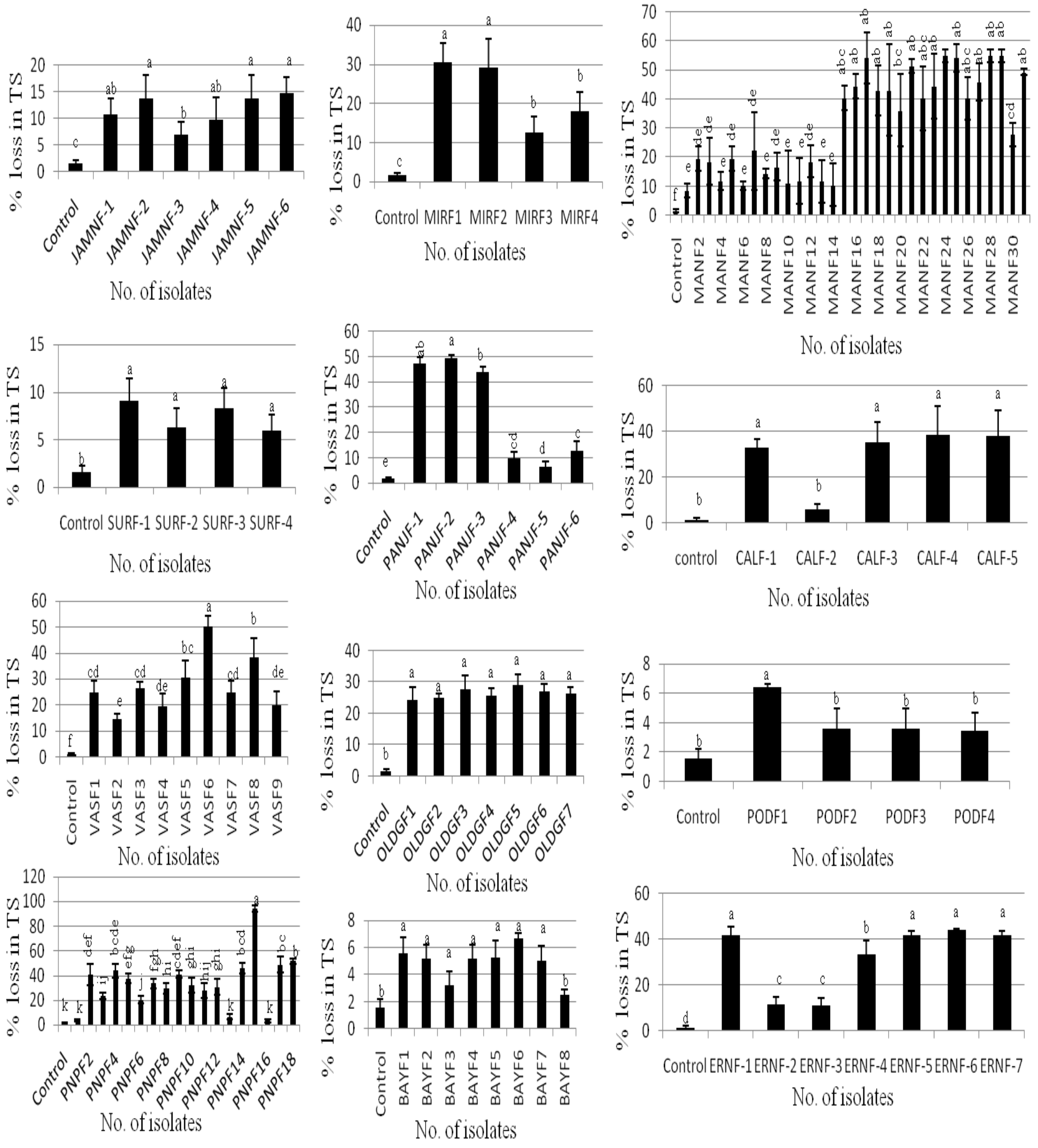

Supplementary Figure S6

Percent loss in TS at pH 3.5 (different lower case letters above the standard deviation bars are significantly different at 0.05 level of significance).

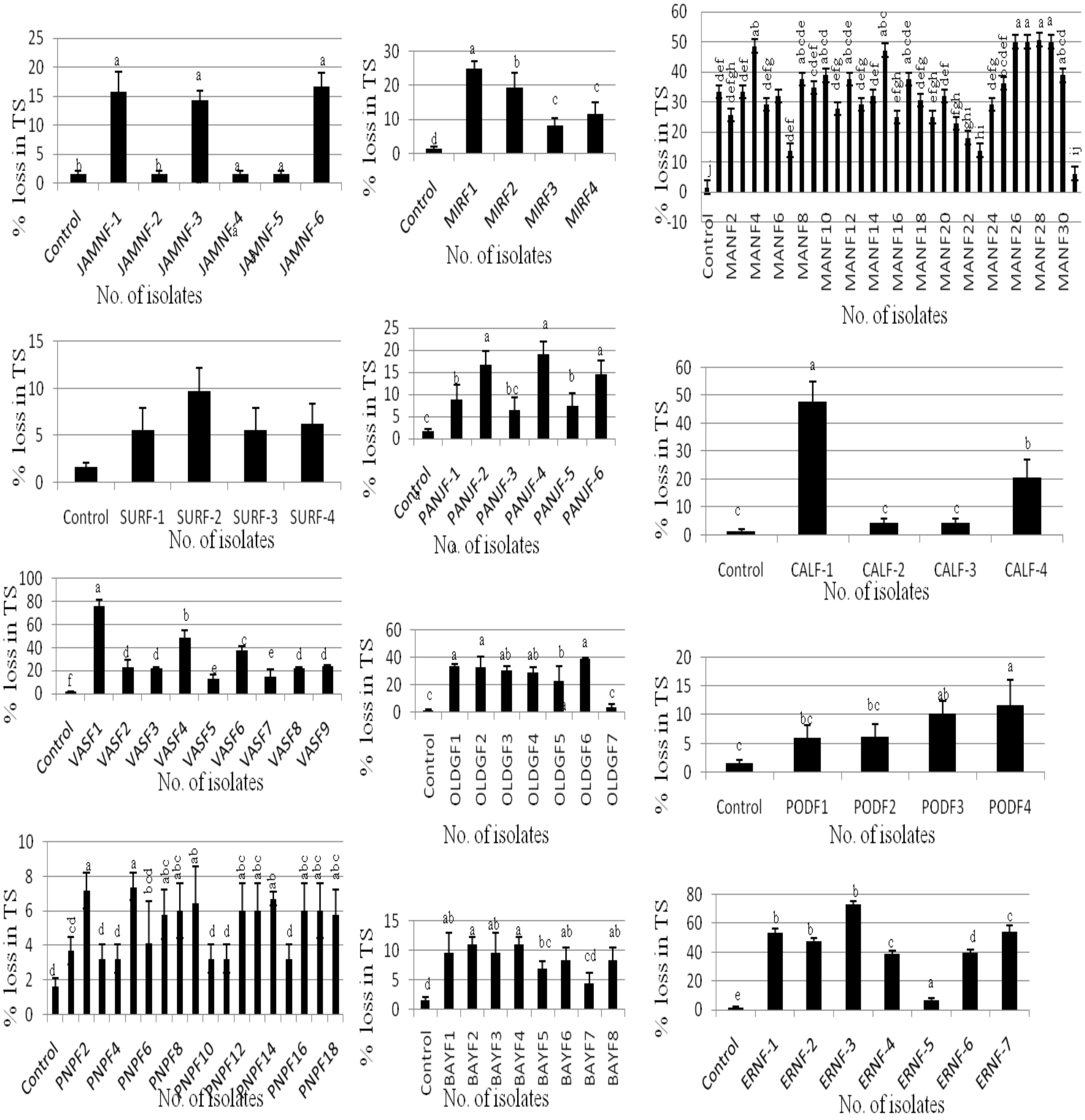

Supplementary Figure S7

Percent loss in TS at pH 7 (different lower case letters above the standard deviation bars are significantly different at 0.05 level of significance).

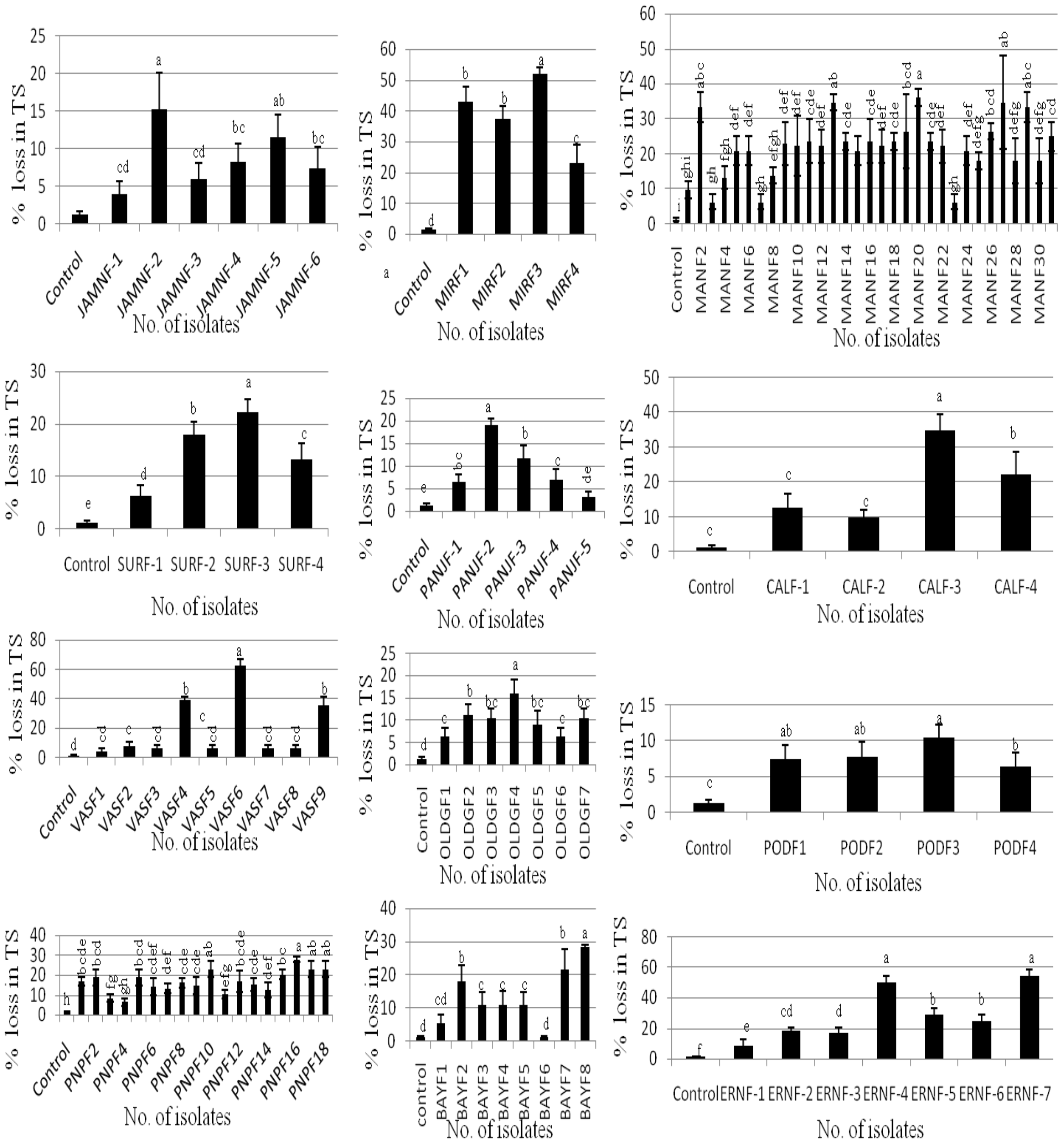

Supplementary Figure S8

Percent loss in TS at pH 9.5 (different lower case letters above the standard deviation bars are significantly different at 0.05 level of significance).

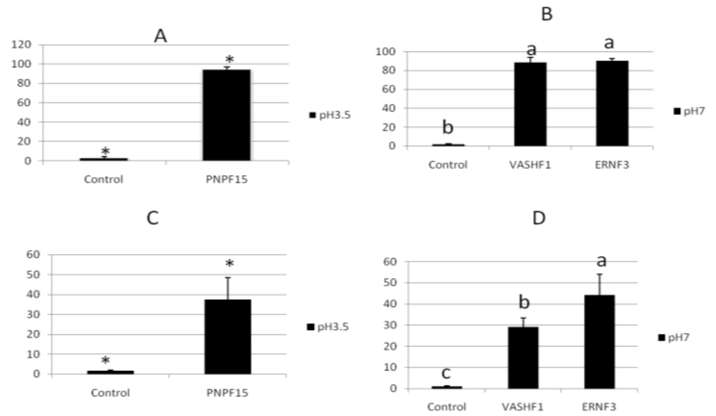

Supplementary Figure S9

Repetition based on % loss in TS: A-B: repetition based on % loss in TS in pretreated PE; C-D: repetition based on % loss in TS in untreated PE (different lower case letters above the standard deviation bars are significantly different at 0.05 level of significance based on ANOVA;*statistically different based on t-test at 0.05 level of significance).

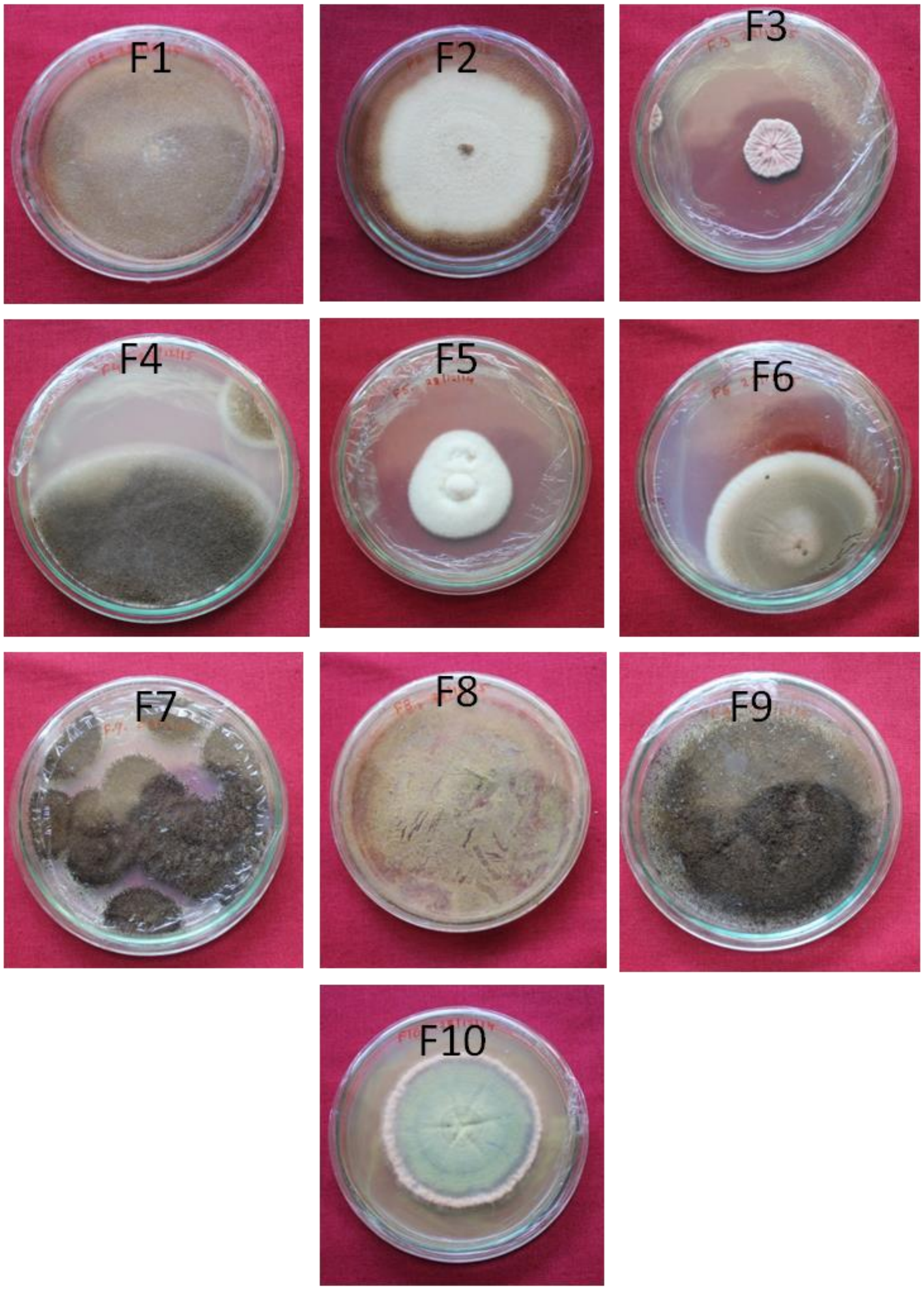

Supplementary Figure S10
Photographs of top 10 most efficient polythene degrading fungi grown on SA plate.

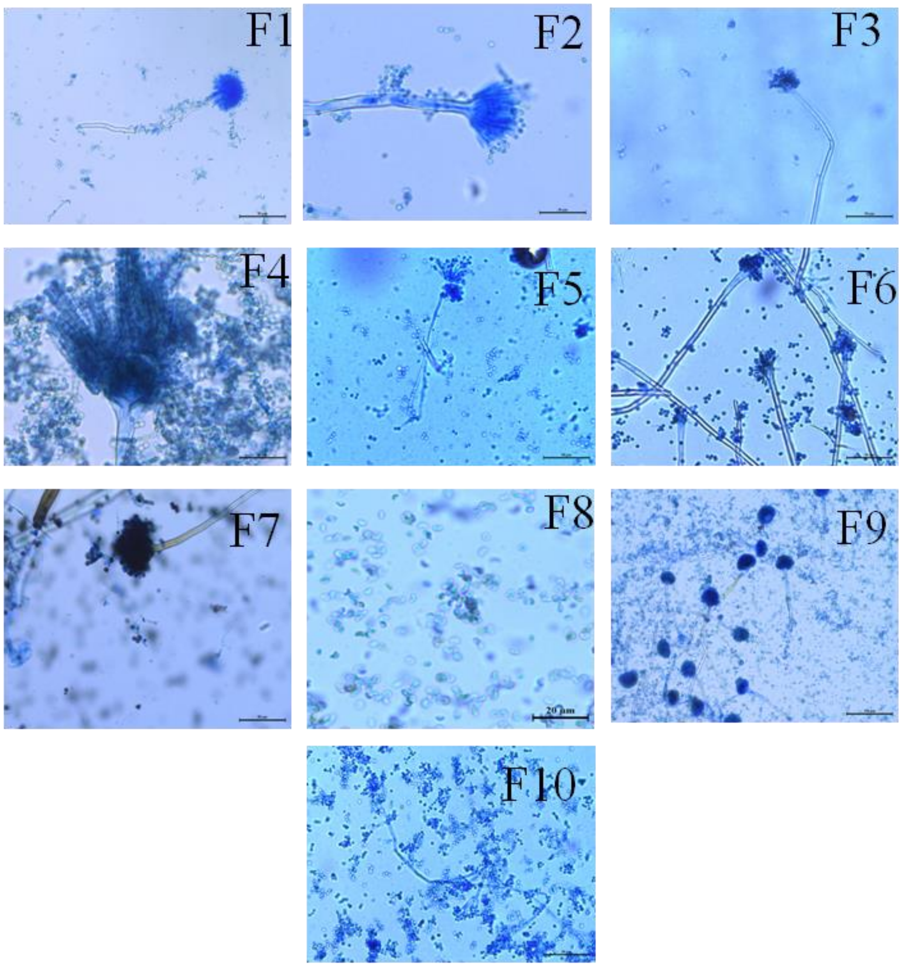

Supplementary Figure S11

Microphotographs of top ten polythene degrading fungi: F1. *Aspergillus* sp. BAYF5/WL; F2: *Aspergillus* sp. MANF1/WL; F3: *Aspergillus* sp. ERNF1/WL; F4: *Aspergillus* sp. BAYF7/WL; F5: *Aspergillus* sp. MANF2/WL; F6: *Aspergillus* sp. PNPF15/TS; F7: *Aspergillus* sp. VASF1/TS; F8: unknown ERNF3/WL; F9: *Aspergillus* sp. VASF6/TS; F10: *Pencillium* sp. MIRF3/TS.

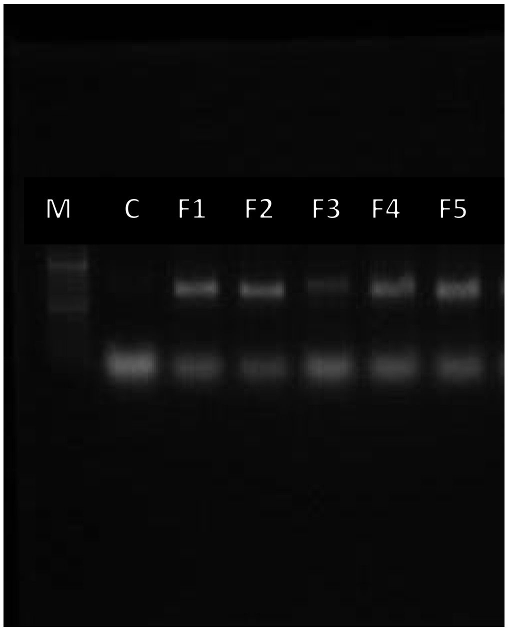

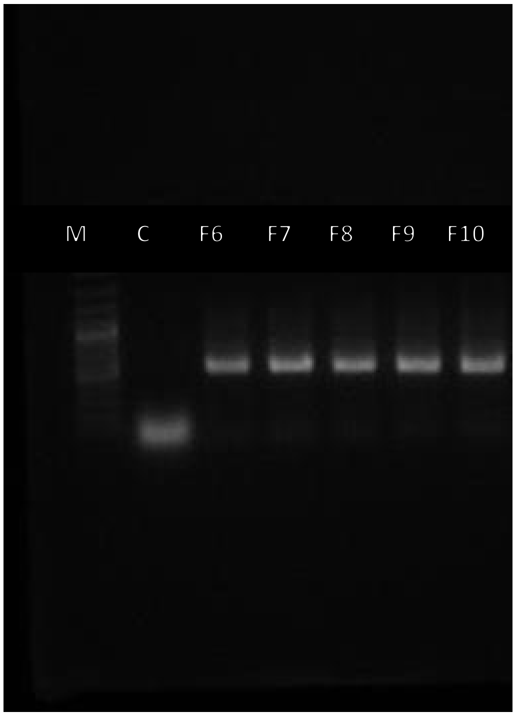

Supplementary Figure S12.
Amplified ITS genes of the polythene degrading fungi separated on 1.2% Agarose gel. M: 100plus DNA ladder, C: negative control; F1: BAYF5; F2: MANGF1; F3: ERNF1; F4 BAYF7; F5:MANGF2; F6: PNPF15; F7: VASF1; F8: ERNF3; F9: VASF6; F10; MIRF3.

Supplementary Table S1. GPS details of the *A. marina* localities along the West Coast of India and locality wise number of the fungal isolates (Reprinted by permission from [Springer Nature]: [Environmental Science and Pollution Research] [Rhizosphere of *Avicennia marina* (Forsk.) Vierh. as a landmark for polythene degrading bacteria, Mohd. Shahnawaz, Manisha K. Sangale, Avinash B. Ade © 2016)

**Sr. No.**	**Locality**	**District**	**State**	**Latitude (N)**	**Longitude (E)**	**Altitude (m)**	**Fungal isolates**
1	New Bedi Port	Jamnagar	Gujarat	22° 54’24.86”	70°03’44.94”	1	6
2	Dumas beach	Surat		21° 08’01.85”	72° 71’51.85”	4.5	4
3	SagarVihar, Vashi	Thane	Maharashtra	19° 04’31.38”	72° 59’02.14”	0	9
4	PNP, Vadkhal	Raigad		18° 41’58.05”	73° 01’34.33”	19	17
5	JakiMirya	Ratnagiri		17° 01’36.56”	73° 16’29.83”	2	4
6	PattoPanjim	North District	Goa	15° 29’57.48”	73° 50’16.73”	3.4	7
7	Old Goa, Diwar			15° 30’36.87”	73° 54’41.93”	0.9	6
8	Bayndoor	Udupi	Karnataka	13° 51’03.77”	74° 36’54.64”	6.4	8
9	Panambur Beach	Mangalore		12° 56’21.63”	74° 48’15.66”	3	31
10	East Kalai	Calicut	Kerala	11° 14’16.60”	75° 47’14.24”	7	6
11	Pudponnani	Malapuram		10° 44’42.47”	75° 56’10.70”	3	4
12	Vallarpadam	Ernakulum		09° 59’26.66”	76° 14’56.96”	8	7
Total							109

Supplementary Table S2. Most efficient polythene degrading fungal isolates based on % WL

Sr. No.	Fungal isolate code	pH	Percent weight loss
1	BAYF5	7	58.51±8.14
2	MANGF1	9.5	41.82±5.47
3	ERNF1	7	37.94±3.06
4	BAYF 7	9.5	30.82±7.58
5	MANGF2	7	28.63±1.65

Supplementary Table S3. Most efficient polythene degrading fungal isolates based on % loss in TS

Sr. No.	Fungal Isolate Code	pH	Percent loss in TS
1	PNPF15	3.5	94.44±2.40
2	VASF1	7	76.04±4.16
3	ERNF3	7	73.26±2.16
4	VASF6	3.5	62.5± 4.16
5	MIRF3	7	54.16±4.16

Supplementary Table S4. Percent change in carbonyl index of the PE strips

Sample code	CI (X)	CI of Control (Y)	Change in CI (Y-X)	% Change in CI Y-X/Y*100
Pretreated PE (Control)	1.025454	1.025454	0.000000	0.000000
MANF1/WL/PT	0.999248	1.025454	0.026206	2.555536
PNP15/TS/PT	0.99846	1.025454	0.026994	2.632383
Untreated PE (Control)	1.044712	1.044712	0.000000	0.000000
MANF1/WL/UT	0.999212	1.044712	0.045500	4.355288
PNP15/TS/UT	0.999366	1.044712	0.045346	4.340556

CI: Carbonyl Index

Supplementary Table S5. Details of sequence submitted to gene bank (NCBI)

Sr. No.	Name of fungi	Fungal isolate code	Gene bank accession number
1	*Aspergillus terreus*	BAYF5/WL	KU551273
2	*Aspergillus terreus*	MANGF1/WL	KU551274
3	*Aspergillus sydowii*	ERNF1/WL	KU551275
4	*Aspergillus niger*	BAYF7/WL	KU551276
5	*Aspergillus versicolor*	MANF2/WL	KU551277
6	*Aspergillus sydowii*	PNPF15/TS	KU551278
7	*Aspergillus awamori*	VASF1/TS	KU551279
8	*Meyerozyma guilliermondii*	ERNF3/TS	KU551280
9	*Aspergillus awamori*	VASF6/TS	KU551281
10	*Penicillium chrysogenum*	MIRF3/TS	KU551282

Supplementary Table S6. Details of ITS primers

Sr. No.	Primer	Sequence	Product size	Annealing temperature
1	ITS5(FP)	GGAAGTAAAAGTCGTAACAAGG	550	59°C
2	ITS4(RP)	TCCTCCGCTTATTGATATGC		
